# Isofagomine *In Vivo* Effects in a Neuronopathic Gaucher Disease Mouse

**DOI:** 10.1371/journal.pone.0019037

**Published:** 2011-04-20

**Authors:** Ying Sun, Huimin Ran, Benjamin Liou, Brian Quinn, Matt Zamzow, Wujuan Zhang, Jacek Bielawski, Kazuyuki Kitatani, Kenneth D. R. Setchell, Yusuf A. Hannun, Gregory A. Grabowski

**Affiliations:** 1 The Division of Human Genetics, University of Cincinnati College of Medicine, Cincinnati, Ohio, United States of America; 2 The Division of Pathology and Laboratory Medicine, Cincinnati Children's Hospital Medical Center, University of Cincinnati College of Medicine, Cincinnati, Ohio, United States of America; 3 The Department of Pediatrics, University of Cincinnati College of Medicine, Cincinnati, Ohio, United States of America; 4 The Department of Biochemistry and Molecular Biology, Medical University of South Carolina, Charleston, South Carolina, United States of America; 5 The Department of Clinical Laboratory, Tottori University Hospital, Tottori University, Yonago, Japan; Baylor Research Institute, United States of America

## Abstract

The pharmacological chaperone, isofagomine (IFG), enhances acid β-glucosidase (GCase) function by altering folding, trafficking, and activity in wild-type and Gaucher disease fibroblasts. The *in vivo* effects of IFG on GCase activity, its substrate levels, and phenotype were evaluated using a neuronopathic Gaucher disease mouse model, 4L;C* (V394L/V394L + saposin C-/-) that has CNS accumulation of glucosylceramide (GC) and glucosylsphingosine (GS) as well as progressive neurological deterioration. IFG administration to 4L;C* mice at 20 or 600 mg/kg/day resulted in life span extensions of 10 or 20 days, respectively, and increases in GCase activity and protein levels in the brain and visceral tissues. Cerebral cortical GC and GS levels showed no significant reductions with IFG treatment. Increases of GC or GS levels were detected in the visceral tissues of IFG treated (600 mg/kg/day) mice. The attenuations of brain proinflammatory responses in the treated mice were evidenced by reductions in astrogliosis and microglial cell activation, and decreased p38 phosphorylation and TNFα levels. Terminally, axonal degeneration was present in the brain and spinal cord from untreated and treated 4L;C* mice. These data demonstrate that IFG exerts *in vivo* effects by enhancing V394L GCase protein and activity levels, and in mediating suppression of proinflammation, which led to delayed onset of neurological disease and extension of the life span of 4L;C* mice. However, this was not correlated with a reduction in the accumulation of lipid substrates.

## Introduction

Gaucher disease is caused by mutations in *GBA1* that encodes acid β-glucosidase (glucocerebrosidase, GCase, EC3.2.1.45). The resultant defective GCase leads to accumulation of the substrates, glucosylceramide (GC) and glucosylsphingosine (GS) [1]. The disease has three clinical variants: Type 1 is primarily a visceral and nonneuropathic disease whereas types 2 and 3 are a continuum of neuronopathic and visceral diseases ranging from acute (type 2, infantile) to subacute (type 3) progressive CNS degenerative diseases [1], [2].

Enzyme therapy with regular infusions of mannose-terminated recombinant human enzymes (imiglucerase, Genzyme; velaglucerase alfa, Shire) has become the standard-of-care for significantly involved type 1 patients [3]. The effects on liver, spleen, hematologic parameters, and bone are dose dependent [4], [5]. Children show excellent responses in growth and bone disease to enzyme therapy [6]. Alternatively, reductions in GC levels can be achieved by partial inhibition of GC synthase, an essential enzyme in the biosynthesis of complex glycosphingolipids [7], i.e. substrate reduction therapy (SRT). SRT with N-butyldeoxynojirimycin (miglustat) shows improvement in some Gaucher disease parameters [8]. Eliglustat Tartrate {(1R,2R)-Octanoic acid [2-(2′,3′-dihydro-benzo [1], [4] dioxin-6′-yl)-2-hydroxy-1-pyrrolidin-1-methyl-ethyl]-amide-L-tartaric acid salt}, an analog of 1-phenyl-2-decanoylamino-3-morpholino-1-propanol (PDMP), is a novel potent inhibitor (IC_50_∼24 nM) of GC synthase. This compound significantly reduces GC accumulation in visceral tissues and cells in the Gaucher disease mouse model [9], [10]. In addition, phase 2 studies in humans show therapeutic effects that mimic those of higher dose enzyme therapy [11]. However, the compound does not reach significant levels in the brain since it is a pGP substrate. [9].

Directly modulating mutant enzyme activity by small molecules was proposed as an attractive alternative approach to treat Gaucher disease using pharmacological chaperones [12], [13]. Competitive inhibitors specific for such enzymes could stabilize mutant enzyme proteins in endoplasm reticulum, assist the enzyme trafficking to lysosome, and restoring the activity [13], [14]. IFG, a potent inhibitor of GCase, is an effective *in vitro* pharmacological chaperone that can enhance selected mutant GCases in fibroblasts [14], [15]. IFG treatment of human patient fibroblasts facilitates mutant enzyme trafficking to lysosome and enhances protein and activity levels for the mutant GCases N370S, V394L, and L444P [14], [15], [16].

Here, IFG was evaluated *in vivo* in a neuronopathic GCase deficient mouse model [17] for its effect on mutant GCase activity, substrate levels, and phenotypic improvement.

## Results

### Treatment of 4L;C* mice with IFG

**Figure 1:**
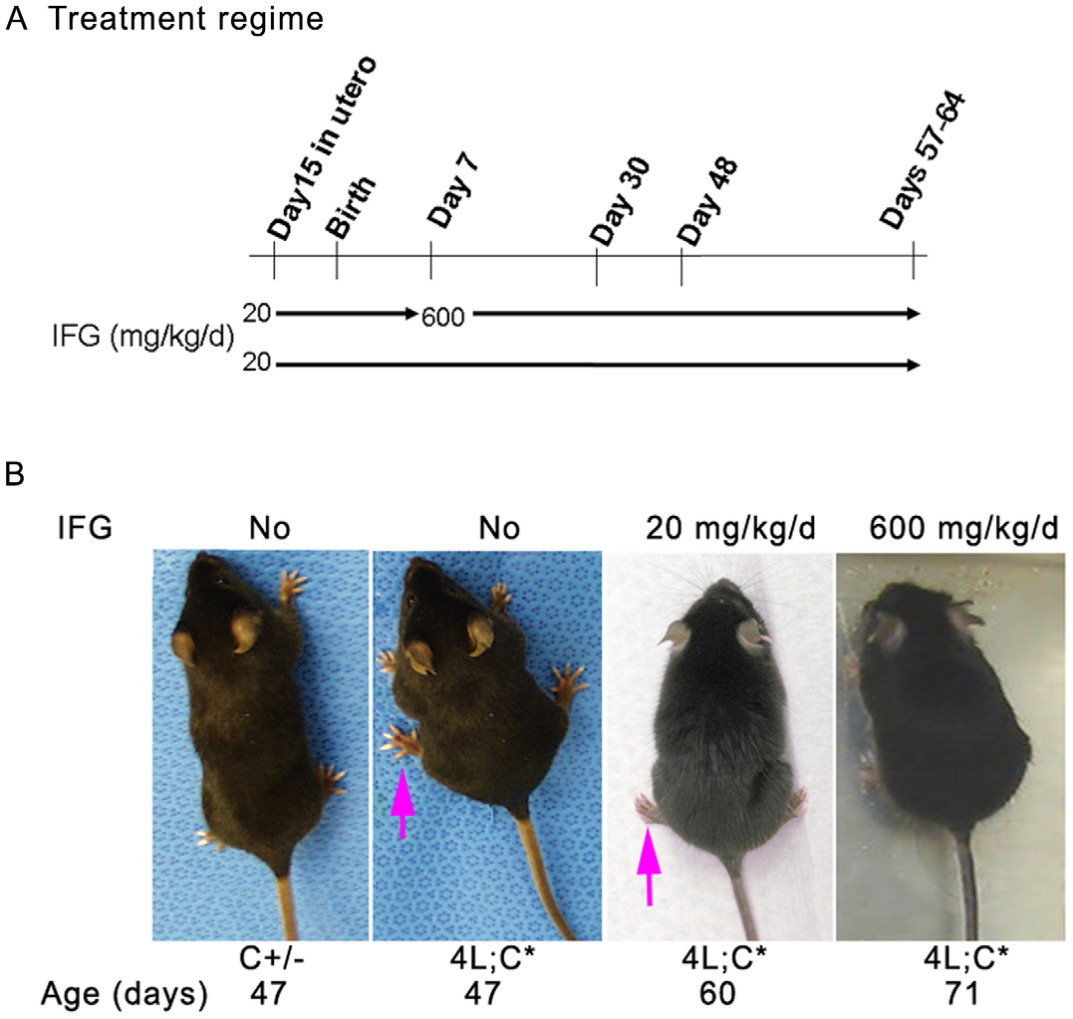
Treatment and phenotypes. (A) IFG treatment regime. IFG was given to pregnant females 3 to 5 days prior birth at 20 mg/kg/d. The first cohort was continued on 20 mg/kg/d for entire course. The dose for the second cohort was increased to 600 mg/kg/d at postnatal 7days. (B) Phenotypes of IFG treated 4L;C* mice. The mice with 20 mg/kg/d IFG showed delayed duck-walk gait (arrow) as seen in the untreated 4L;C* mice. The mice on 600 mg/kg/d IFG showed spastic walk.

**Figure 2:**
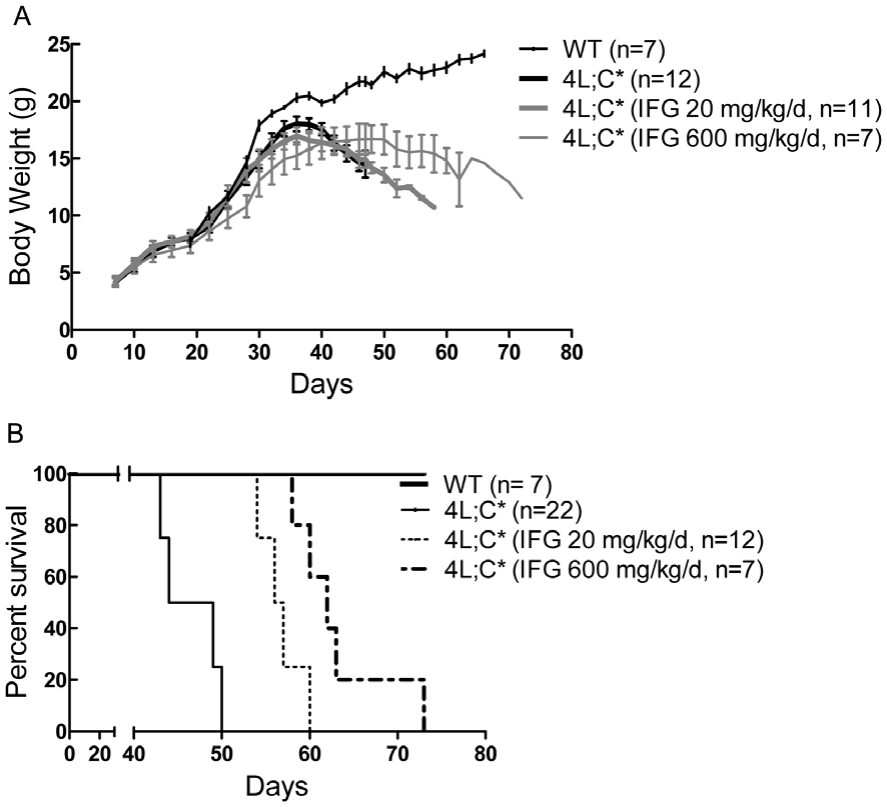
Life span and body weight. (A) Both treated and untreated 4L;C* mice had normal growth of their body weight till 35 days. The body weight of untreated 4L;C* mice started to decline at ∼35 days. The mice on 20 mg/kg/d IFG showed decrease of body weight at ∼35 days. The body weight of 4L;C* mice treated by 600 mg/kg/d IFG started decreasing at ∼48 days. Their body weight grew slower than untreated 4L;C* mice. (B) The mice in both cohorts had significantly extended life span to average 54 days (p = 0.0002) by 20 mg/kg/d or 63 days (P<0.0001) by 600 mg/kg/d IFG treatment relative to the untreated mice (∼48 days). The Kaplan-Meier survival curves were analyzed using the Mantel-Cox Test.

The *in vivo* effects of IFG were tested using 4L;C* mice that develop neurological signs, including a duck-like waddling gait at ∼30 days, and then die from progressive CNS disease by ∼48 days. Three to five days prior birth, IFG was added to the drinking water of pregnant dames in a dose calculated to provide ∼20 mg/kg/d based on measured water intake (see methods). At postnatal day 7 (P7), one cohort was maintained on 20 mg/kg/d IFG and a second cohort on 600 mg/kg/d (Fig. 1A). The body weights (BW) of the mice were recorded from P7 to terminal stage. The 4L;C* mice receiving 20 mg/kg/d IFG showed normal increases of BW to ∼35 days and then declined to ∼70% of WT levels at terminal stage (Fig. 2A). The trend of BW change was similar to that observed with untreated 4L;C* mice. These treated 4L;C* mice developed the characteristic duck-walk gait at 40-44 days and their life span was 54 days on average or about 6–10 days longer than untreated mice (Fig. 2B). The 4L;C* mice receiving 600 mg/kg/d showed slow decreases in BW after 50 days (Fig. 2A). They did not develop duck-walking, but showed spastic-like gait starting 47–50 days (Fig. 1B). The average life span was 63 days or about 15–18 days longer than controls, or ∼34% increase in lifespan (Fig. 2B). The untreated and 20 mg/kg/d IFG treated 4L;C+/− mice showed comparable BW attainments to WT and exhibited a normal phenotype during the entire study period. In comparison, 4L;C+/− mice treated with 600 mg/kg/d IFG showed slightly lower body weight (data not shown). The untreated 4L;C* mice are infertile and male 4L;C* mice treated with 20 or 600 mg/kg/d IFG produced only a single litter.

Additional regimes of IFG treatment were tested. The 4L;C* mice were treated with IFG in a 3-day-on/4-day-off trial. These mice received IFG starting 3-days prior birth at 20 mg/kg/d as above. Then, IFG was stopped from P3 to P7 (4-days off) and then it was given at 20 mg/kg/d for the next 3 days (3-day on). The cycle was continued until the end stage. The mice on this 3-day- on/4-day-off schedule showed no differences of phenotype, onset of signs, or life span (55 days) compared to the mice receiving 20 mg/kg/d of uninterrupted treatment. If IFG treatment was started on postnatal day 25 at 600 mg/kg/d, there was less of an effect on the life span of 4L;C* mice (54 days) compared to starting the same dose prior to birth (63 days). In 5 litters in which IFG (600 mg/kg/d) was begun at 3–5 days prior birth, all such pups died on postnatal days 1 to 3. The pups in one litter survived when this treatment started 2 days prior to birth. The results indicate that earlier treatment started had better efficacy, but that non-toxic doses were needed before the greater benefit of higher doses could be observed; 600 mg/kg/d extended the life span compared 20 mg/kg/d, and the administration of 600 mg/kg/d prior to birth is toxic to the pups.

### IFG enhanced GCase activity and protein

**Figure 3:**
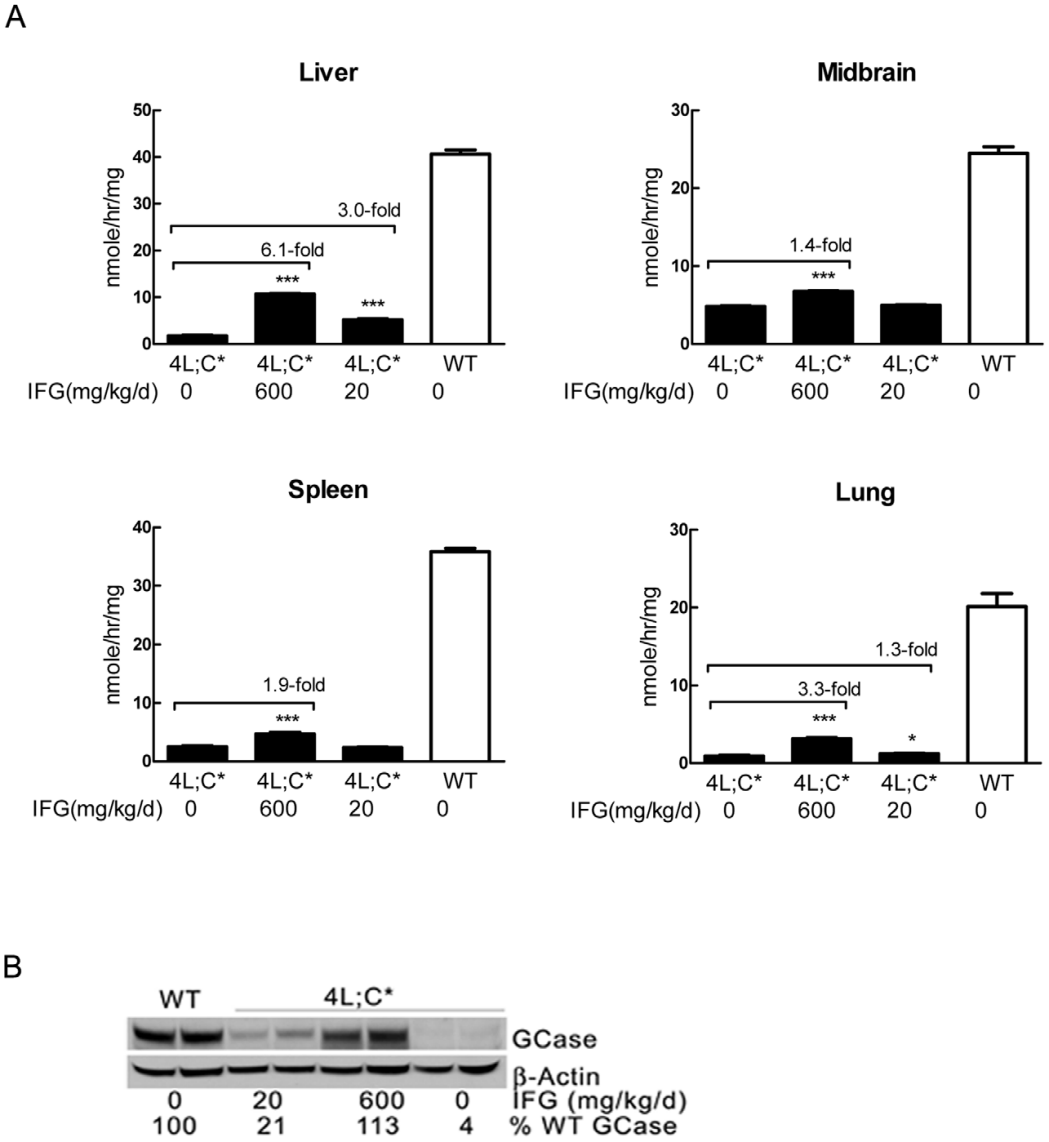
GCase activity and protein. (A) GCase activity was significantly increased in the treated 4L;C* liver by 3-fold in 20 mg/kg/d or 6-fold in 600 mg/kg/d IFG treatment groups, respectively. In the midbrain GCase was significantly increased by 1.4-fold in 4L;C* mice treated with 600 mg/kg/d IFG, but not changed with 20 mg/kg/d IFG compared to the untreated group. The GCase activity in the 4L;C* spleen was enhanced by 600 mg/kg/d IFG, whereas both 20 mg and 600 mg/kg/d IFG increased GCase activity in the lung. (B) GCase protein was increased in the liver of IFG (20 or 600 mg/kg/d) treated 4L;C* mice. GCase protein were determined by immunoblot analyses using anti-mouse GCase antibody and normalized to β-actin signal in the same sample. The levels were presented as percentage relative to GCase in WT mice. All the tissues used in GCase activity and protein analyses were from 28 days old mice. The data represent the mean±S.E. for three mice assayed in triplicate and analyzed by Student's t-test. ***, p<0.001.

The mutant GCase activity and protein levels in the tissues were analyzed in 4L;C* mice (Fig. 3). In the liver, IFG administration enhanced the mutant GCase activity by 3-fold at 20 mg/kg/d and by 6-fold at 600 mg/kg/d compared to that in untreated 4L;C mice. The 6-fold increase of V394L GCase activity corresponded to ∼25% of WT levels, and was similar to the maximal effect observed in cultured skin fibroblasts from V394L/V394L or 4L;C* mice (data not shown). On 600 mg/kg/d, the mutant GCase activity was increased to a lesser extent in midbrain (1.4-fold) and spleen (1.9-fold). Lung mutant GCase activity was increased by 3.3-fold or 1.3-fold in the mice treated with IFG at 600 or 20 mg/kg/d, respectively. Immunoblot analyses using a specific anti-mouse GCase antibody showed that IFG increased GCase protein levels in the liver to WT levels (600 mg/kg/d) and to 21% of WT levels (20 mg/kg/d). In liver, these respective increases in GCase protein were about 28- or 5-fold compared to untreated 4L;C* (Fig. 3B). The results indicated a discrepancy between the activity and protein increases, and indicate that the IFG *in vivo* effect on activity was either inefficient or inhibitory. Also, tissue specific effects were observed with the liver enzyme having a greater response to IFG than that in the brain, spleen and lung. Whether this is due to enzyme differences in the various tissues or, more likely, IFG tissue distribution is unknown.

### Substrate levels in IFG treated mice

**Figure 4:**
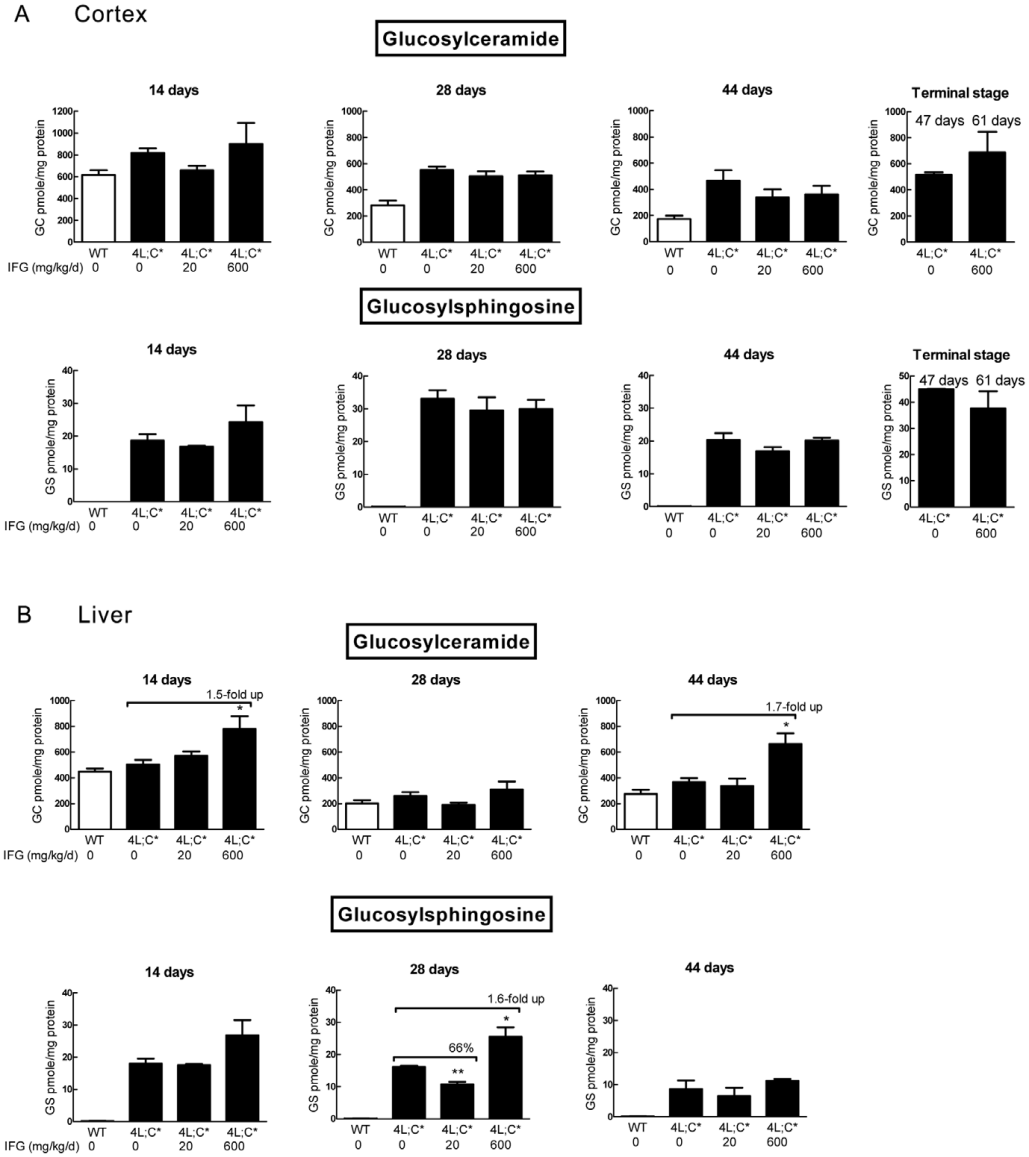
Analyses of GC and GS levels in the cortex and liver by LC/MS. (A) Cortex GC and GS levels in IFG treated 4L;C* did not show significant reduction relative to the untreated mice. GC and GS levels remained accumulated at terminal stage in 600 mg/kg/d IFG treated 4L;C* brain. (B) Increased liver GC were detected in 600 mg/kg/d IFG treated 4L;C* mice at 14 and 44 days relative to untreated 4L;C* liver. GS level decreased in 20 mg/kg/d IFG treated 4L;C* liver at 28 days. GS level in 600 mg/kg/d IFG treated 28 day liver were higher than untreated 4L;C* liver. The data represent the mean±S.E. and analyzed by Student's t-test. *, p<0.05 (n = 3 mice). The GC and GS levels were normalized by protein level in the tissues lysate.

**Figure 5:**
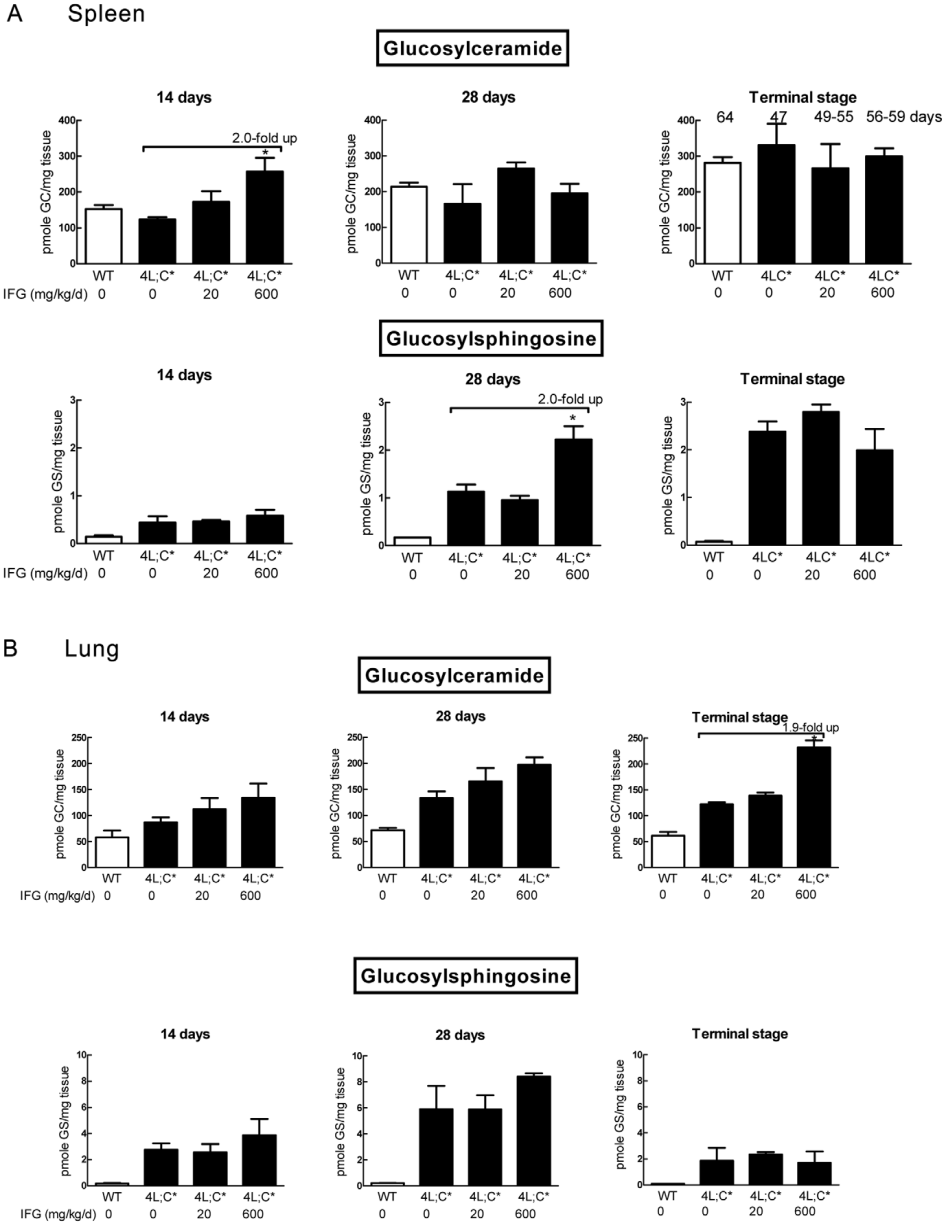
Analyses of GC and GS levels in the spleen and lung by LC/MS. (A) Spleen GC level in 4L;C* mice was not different from that in the WT. GS level was accumulated in 4L;C* spleen. By 600 mg/kg/d IFG treatment, GC or GS were increased by 2-fold in 4L;C* spleen at 14 day or 28 day, respectively. (B) GC and GS were accumulated in 4L;C* lung. IFG treatment did not reduce the substrate level in 4L;C* lung. Increased GC level was in 600 mg/kg/d IFG treated lung at terminal stage (Age was same as spleen in Fig. 5A). The data represent the mean±S.E. and analyzed by Student's t-test. *, p<0.05 (n = 3 mice). The GC and GS levels were normalized by tissue weight.

GC and GS levels in 4L;C* tissues were determined by LC/MS. The tissues were collected from IFG treated (20 and 600 mg/kg/d) 4L;C*, untreated 4L;C* and WT mice at 14, 28, 44 days, and at terminal stages. Compared to untreated 4L;C*, GC and GS levels in various tissues were either unchanged or increased (Fig. 4 and Fig. 5). The longer treatments with 600 mg/kg/d led to GC and/or GS increases in visceral tissues (Fig. 4B and Fig. 5). The cerebral cortical levels were not significantly different in treated mice (Fig. 4A). At terminal stages, GC and GS levels in 600 mg/kg/d IFG treated brains were increased over baseline (Fig. 4A).

**Figure 6:**
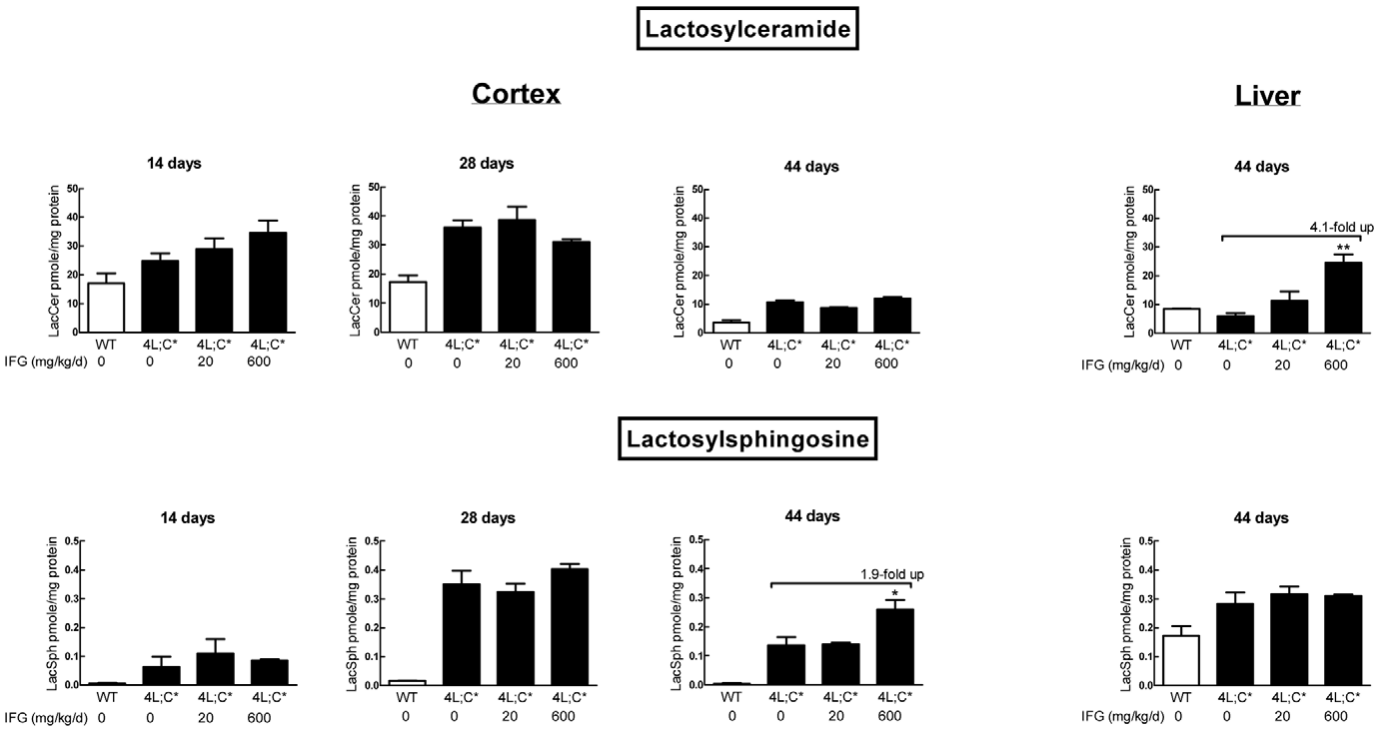
Analyses of lactosylceramide and lactosylsphingosine levels by LC/MS. Lactosylceramide (LacCer) level was not decreased in IFG treated 4L;C* cortex relative to the untreated mice. Increased lactosylsphingosine (LacSph) was detected in 600 mg/kg/d IFG treated cortex at 44 days. In the liver, LacSph level was not altered, but LacCer was increased in 600 mg/kg/d IFG treated mice at 44 days. The data represent the mean±S.E. and analyzed by Student's t-test. **, p<0.01; *, p<0.05 (n = 3 mice). The GSL levels were normalized by protein level in the tissues lysate.

Cerebral cortical lactosylceramide (LacCer) and lactosylsphingosine (LacSph) levels in 4L;C* mice were not reduced by IFG treatment (Fig. 6). In general, LacCer and LacSph levels were either increased or unchanged in 600 mg/kg/d IFG treated mice compared to either untreated or to 20 mg/kg/d treated mice. Ceramide levels in 4L;C cortex and liver were at WT levels and were not altered by IFG treatment (data not shown). These data indicated that GC and GS levels were not substantially reduced in IFG treated 4L;C* mice. High dose (600 mg/kg/d) IFG caused accumulation of GC or GS in selected visceral tissues.

**Figure 7:**
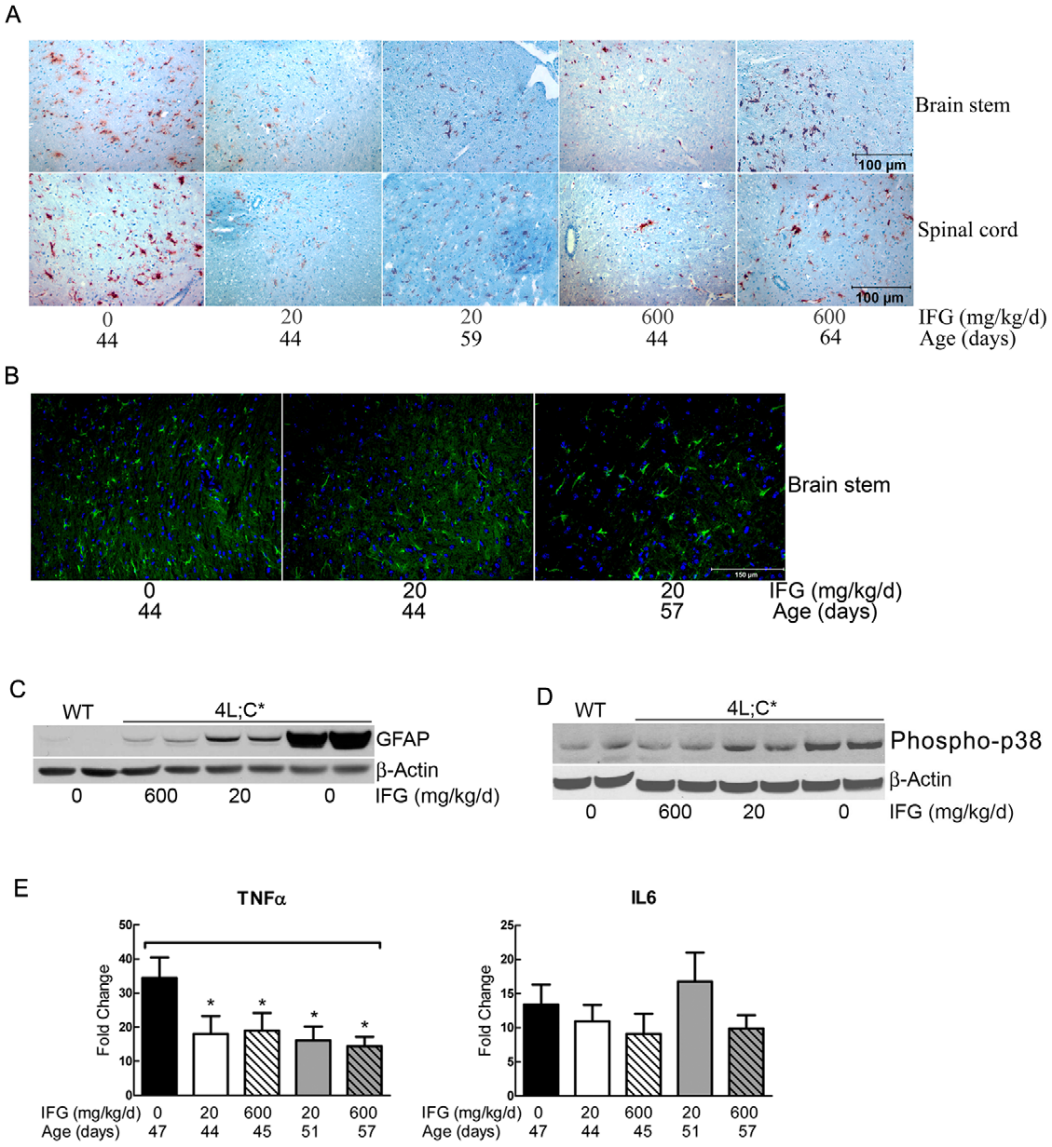
Proinflammation. (A) The brain stem and spinal cord sections were stained with anti-CD68 antibody, a macrophage-like marker. Positive CD68 signals (brown) on microglial cells demonstrated proinflammation. IFG (20 or 600 mg/kg/d) treated 4L;C* showed decreases of proinflammation at 44 days and increases of proinflammation at 59 or 64 days (terminal stage). (B) Enhanced GFAP staining (green) was in untreated 44 days old 4L;C* brain stem. IFG (20 mg/kg/d) treated 4L;C* showed decreases of GFAP signal at 44 days. GFAP signal were increased at terminal stage at 57 days. (C) GFAP protein in 44 days midbrain tissues was determined by immunoblot. Compared to untreated 4L;C*, GFAP levels were decreased in 20 mg/kg/d IFG and further reduced in 600 mg/kg/d IFG treated midbrain. (D) Phospho-p38 in 44 days midbrain tissues was analyzed by immunoblot. Increased Phospho-p38 was in untreated 4L;C* midbrain relative to WT. Phospho-p38 level was decreased in 20 mg/kg/d IFG treated midbrain and nearly to WT level in 600 mg/kg/d IFG treated mice. (E) TNFα and IL-6 mRNA in midbrain was determined by qRT-PCR. TNFα and IL-6 mRNA levels were increased by 35- and 13-fold in the untreated 4L;C* midbrain, respectively. In IFG (20 or 600 mg/kg/d) treated midbrain, TNFα mRNA were reduced at 44 or 45 days and at terminal stage (51 or 57 days). IL-6 mRNA level was not altered by IFG treatment. The data represent the mean±S.E. and analyzed by Student's t-test (n = 3 mice, assayed in duplicate).

### IFG suppressed proinflammation

Proinflammation is a pathologic response in 4L;C* brain as demonstrated by microglial cell activation and astrogliosis [17]. IFG (20 or 600 mg/kg/d) treated 4L;C* showed decreased CD68 staining of microglial cells at 44 days, indicating suppression of microglial cell activation (Fig. 7A). With the disease progressing to the terminal stages, the CD68 positive microglial cells increased in both dosage groups (Fig. 7A). GFAP signals were increased, indicating astrogliosis. In the 20 mg/kg/d IFG treated 4L;C* brain stem, GFAP signals were weak at 44 days and increased by 57 days, i.e., terminal stage (Fig. 7B). Immunoblot analyses of GFAP of 44 day midbrain samples showed the reduction of GFAP level in IFG treated mice (Fig. 7C). High dose IFG (600 mg/kg/d) was more effective in reducing GFAP signal levels than the lower dose in age matched samples.

p38 is involved in mediating inflammatory cytokine expression [18], [19]. By immunoblotting with anti-Phosphorylated/active-p38 antibody, decreased phospho-p38 levels were detected in IFG treated 4L;C* midbrain, and the effect was dose dependent (Fig. 7D). The mRNA levels of p38α, β, γ, and δ were not significantly altered in the treated and untreated 4L;C* relative to WT, as determined by qRT-PCR analyses (data not shown). TNFα and IL6 are up-regulated during inflammation. By qRT-PCR analyses, increased TNFα and IL-6 mRNA levels were found in untreated 4L;C* midbrains (Fig. 7E) and brain stems (data not shown). TNFα levels were reduced by ∼50% in IFG treated mice at 44 days and remained at decreased levels until the terminal stage (51 or 57 days). IL-6 mRNA levels remained elevated during IFG treatment with either dose at 44 days and at terminal stages. These results suggest that IFG attenuates the proinflammatory responses of microglial cells and astrocytes in 4L;C* brain.

### Histology

**Figure 8:**
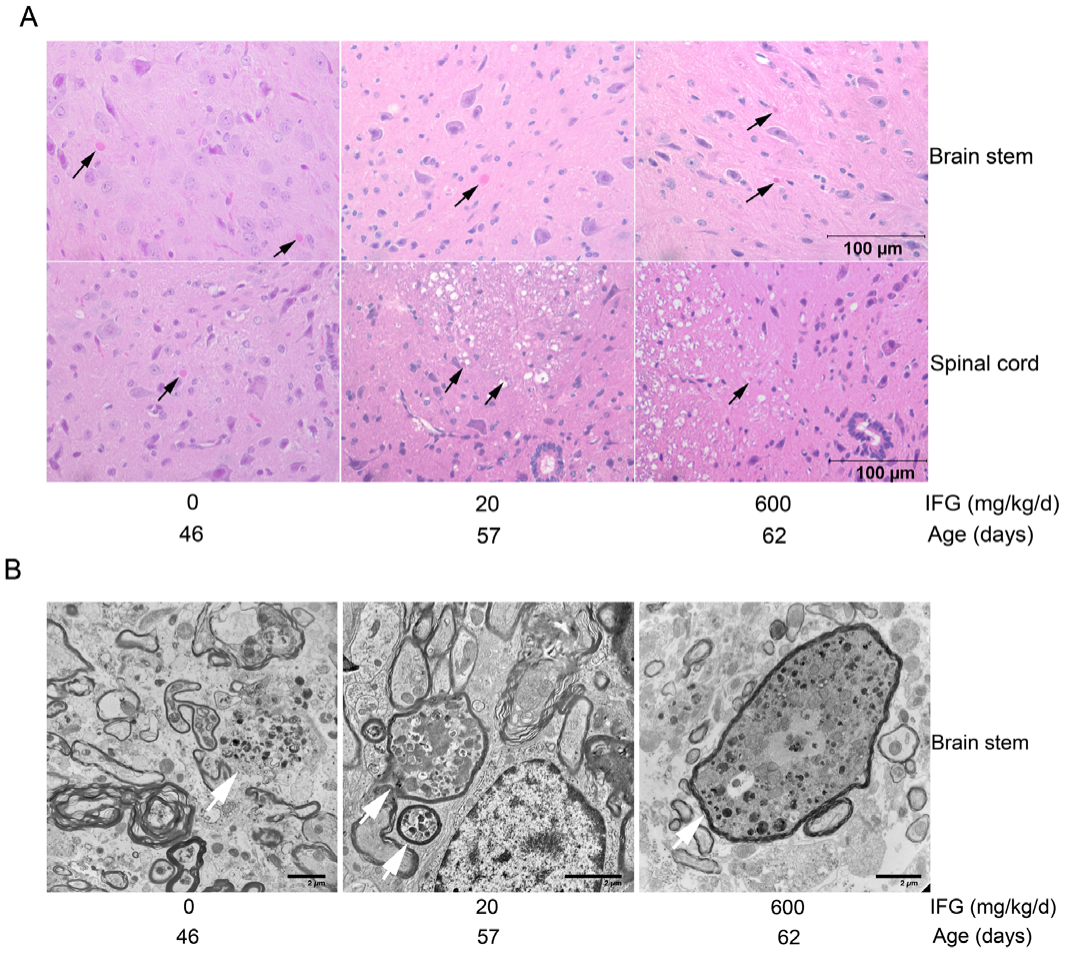
Histopathology. (A) H&E stained brain stem and spinal cord sections showed axonal degeneration (arrow) in untreated 4L;C* and 4L;C* mice treated by 20 or 600 mg/kg/d IFG. The tissues were collected at terminal stage. (B) Electron microscopy of brain stem. Axonal inclusions (arrow) in brain stem were present in untreated 4L;C* (left panel) at 43 days, 20 mg/kg/d IFG treated 4L;C* mice at 55 days (middle panel) and 600 mg/kg/d IFG treated 4L;C* mice at 62 days (right panel).

The 4L;C* mice display axonal degeneration in the brainstem and spinal cord [17]. Axonal degeneration was found by H&E staining of brain stem and spinal cord sections from the 4L;C* mice untreated or treated with either 20 or 600 mg/kg/d IFG (Fig. 8A). Ultrastructural studies showed axonal inclusions in both treated and untreated 4L;C* mouse brain stems (Fig. 8B), midbrain and spinal cord (data not shown). These findings show that IFG treatment did not fundamentally change the brain pathology in 4L;C* mice.

## Discussion

Pharmacological chaperones are a class of small molecules that enhance enzyme stability and can assist in trafficking of mutant enzymes from endoplasmic reticulum to normal destination such as the lysosome for GCase. IFG is a specific reversible GCase inhibitor (K_i_ ∼20 nM) of WT GCase [20]. The binding of IFG to the active site of GCase locks the enzyme in its substrate-bound conformation and thermodynamically stabilizes the enzyme [21]. IFG binding affinity for GCase is greater (K_i_ ∼<12 nM) at the neutral pH of the ER and lesser (K_i_ ∼>50 nM) at the acidic lysosomal pH (Liou and Grabowski observations). Importantly, IFG enhances mutant activity in fibroblast having the N370S [15], [20] and the L444P GCase in lymphoid lines and fibroblasts [16] (Liou and Grabowski unpublished). Using partially purified N370S GCase, IFG stabilizes the enzyme to thermal denaturation [21] (Liou and Grabowski unpublished). These findings suggest the potential for IFG as a therapeutic mutant enzyme enhancer that either improves enzyme stability or trafficking to the lysosome or both. The latter would facilitate dissociation of the GCase-IFG complex in the lysosome and allowing the GCase to bind its natural substrates. The finding that GCase in IFG treated cells have enhanced activity after dilution of the inhibitor implies that the free GCase retains an enhanced conformation for some time after dissociation. Importantly, orally administered IFG distributes to visceral organs and the brain [16], suggesting an improved delivery compared to enzyme therapy that has little or no delivery to lungs, bone marrow, lymphoid tissues, and brain [22]. However, with these promising *in vitro* assay results, *in vivo* demonstration of improved substrate clearance and therapeutic efficiency remained to be shown.

In this study, a neuronopathic GCase-based mouse model having the V394L mutation was evaluated for IFG's *in vivo* effect on enzyme activity and substrate levels in CNS and visceral organs. This model was generated by crossing V394L homozygous to saposin C null mice. V394L homozygous mutant mice have residual enzyme activity of 4–10% of WT levels that is sufficient to maintain substrate flux and not present major GC or GS accumulation or cause a significant abnormal phenotype [23], [24]. Since saposin C deficiency reduces GCase stability to proteolysis [25], the 4L;C* mice have decreased 4L GCase and develop a major neurological phenotype associated with accumulation of GS and GC in the brain [17]. Importantly, the saposin C-/- mice do not show proinflammation until after 6 months of age in the visceral or brain. In addition, the saposin C-/- mice do not develop any phenotype or pathology until after 10 months of age. In saposin C-/- mouse model, absence of saposin C reduced GCase protein by ∼50% of WT levels and led to activity reductions, but GC and GS levels are not increased in the brain and visceral tissues [26]. The phenotype of 4L;C* mouse model mimics some neuronopathic variants of human Gaucher disease. Here, IFG treatment of 4L;C* mice enhanced the V394L GCase protein and activity levels up to 25% of WT levels in the livers and about 2-fold in the brain. This was not accompanied by significant reductions in the accumulated substrates, GC and GS. Indeed, substrate levels increased under high dose IFG (600 mg/kg/d) treatment. This inhibitory effect of IFG and the mutant GCase was clearly toxic/lethal to developing pups as was evident when pregnant dames were administered this dose. Lower doses (20 mg/kg/d) did not have such toxic effect. To overcome such inhibitory effects, an on/off regimen had been suggested [16]. Here, no differences were found on the phenotype onset or life span between the on/off and continuous treatments. Lacking significant reduction of substrate levels by IFG in this model could be attributed to inhibitory effect on GCase from locally excessive IFG, and/or absence of saposin C's protection.

Despite the lack of IFG effect on the GC and GS levels, the treatment extended 4L;C*'s lifespan by >25%. Importantly, the pathological microglial infiltration in the untreated mice was delayed in the treated mice until the terminal stage. Thus, IFG treatment slowed disease progression, but did not change the ultimate disease outcome. Intriguingly, in the IFG treated mice, proinflammation was suppressed in the middle stage of treatment (44 days). However, the effect was lost in the terminal state and this was not different from the end stage in untreated mice. Such effects on the anti-proinflammatory responses were dose dependent, suggesting specific effect of IFG. Proinflammatory responses have been associated with neurodegeneration of other lysosomal storages diseases [27], and anti-inflammatory treatments attenuated the phenotype and extending lifespan [28], [29], [30]. The molecular targets for IFG to have such anti-proinflammatory effects are unknown. TNFα and IL-6 are the proinflammation cytokines that are dependent on p38 signaling for their production [18], which has implications in neurodegenerative diseases [31], [32]. IL-6 protein and mRNA levels remained elevated during IFG treatment. However, the reductions of brain TNFα and phosporylation of p38 with IFG treatment suggests that these pathways are involved in the IFG mediated proinflammatory attenuation. In cell lines, ceramide generated from GC through the salvage pathway by enhancing GCase activity can reduce p38-mediated decreases in IL-6 levels, which would be anti-inflammatory [19]. In the brain, the total levels of GC and ceramide were not affected by IFG, and, therefore, the IL-6 pathway does not appear functional in this *in vivo* system. However, p38 phosphorylation was significantly reduced suggesting some connection. One intriguing possibility is that while the total levels of GC and ceramide remained the same, their subcellular distribution may have been different. Based on chaperone function, we could speculate that levels of GC and ceramide may have been partly corrected in the target organelle (e.g. lysosome) but not in other compartments.

Alternatively, off-target effects of IFG might be evoked. IFG is an iminosugar that is a member of the class of inhibitors for glycosidases and glycosyltransferases. Iminosugars have therapeutic applications in the diseases associated with the metabolism of glycoconjugates to treat tumor metastasis, influenza virus infection, and lysosomal storage diseases [33], [34]. Single oral dose of 600 mg/kg to the rat achieves IFG concentration of 1.7 µM in the brain [16]. The continuous dosing of IFG (20 and 600 mg/kg/d) used here could have achieved much higher concentration in the brain than the K_i_ for GCase (20 nM). At such high concentrations, IFG could inhibit other glycosidase involved in inflammatory pathways, e.g. chitinase [35].

This study revealed the importance of *in vivo* analyses of the effects of pharmacological small molecules on mutant enzyme's function. Clearly, the ultimate effects on substrate reductions are essential for GCase or other lysosomal enzymes. Thus, *ex vivo* results of increasing GCase activity and protein by chaperones are insufficient to conclude that *in vivo* substrate degradation will occur and need to be interpreted cautiously when evaluating additional new compounds.

## Materials and Methods

### Materials

The following were from commercial sources: NuPAGE 4–12% Bis-Tris gel, NuPAGE MES SDS running buffer (Invitrogen, Carlsband, CA). 4-methyl-umbelliferyl-β-D-glucopyranoside (4MU-Glc; Biosynth AG, Switzerland). Sodium taurocholate (Calbiochem, La Jolla, CA). M-PER Mammalian Protein Extraction Reagent and BCA protein Assay reagent (Pierce, Rockford, IL). Hybond™-ECL™ nitrocellulose membrane and ECL detection reagent (Amersham Biosciences, Piscataway, NJ). Anti-fade/DAPI, Methyl green, ABC Vectastain and Alkaline phosphatase kit II (Black) (Vector Laboratory, Burlingame, CA). Rat anti-mouse CD68 monoclonal antibodies (Serotec, Oxford, UK). Mouse anti-GFAP monoclonal antibody (Sigma, St. Louis, MO). Rabbit anti-active p38 polyclonal antibody (Promega, Madison, WI). IFG tartrate was provided by Amicus Therapeutics (Cranbury, NJ).

### Animal care and drug treatment

4L;C* mice generation was as described [17]. Briefly, 4L;C* (V394L/V394L, saposin C-/-) mice were obtained from cross breeding saposin C+/-, V394L/V394L male and female mice. The strain background of 4L;C* was C57BL/6J/129SvEV. *In utero* IFG treatment was accomplished by providing the pregnant dames drinking water containing the requisite amount of IFG for 3–5 days prior to birth. In addition, mothers were kept on IFG during the weaning period so that the pups had continuous exposure to IFG via breast milk. Following weaning the mice were administrated IFG in drinking water under various specified regimens. Average daily water intake for each mouse is ∼5 ml. At postnatal day 7, cohorts were treated with IFG either 600 or 20 mg/kg/d. Body weight, cage activity, and neurological phenotypes of the mice were monitored daily. The mice were maintained in microisolators in accordance with institutional guidelines under Institutional Animal Care and Use Committee (IACUC) approval at Cincinnati Children's Hospital Research Foundation (CCHRF). IACUC at CCHRF approved the study described in this manuscript with Animal Use Protocol number 8D10085. Other dosing schemes were as indicated in the text.

### Histology and immunohistochemistry

After being anesthetized, the mice were perfused with saline followed by 4% paraformaldehyde prior tissues dissection. The tissues fixed in 10% formalin were processed for paraffin sections and stained with hematoxylin and eosin (H&E). Paraffin sections of brain and spinal cords were stained with mouse anti-GFAP as an astrocyte marker. Biotinylated goat anti-mouse IgG and streptavidin conjugated fluorescent 480 (green) were applied to the sections. DAPI (blue) in antifade was used for cell nuclei staining. Tissues fixed in 4% paraformaldehyde were processed for frozen blocks. Frozen sections were stained with rat anti-CD68 monoclonal antibody [36]. Methyl green was used for cell nuclei staining. Karnovsky's fixative was used for ultrastructural studies.

### Glycosphingolipid analyses

Glycosphingolipids (GSLs) in the brain and liver lysates were extracted and analyzed by LC/MS at the Lipidomic Core at the Medical University of South Carolina [37]. GC and galactosylceramide in brain were separated for the LC/MS analyses. GC and GS levels in the tissue were normalized to protein content in the lysate.

GSLs in spleen and lung tissue (5 mg) were extracted [36], and GC and GS analyses were carried out at CCHRF by ESI-LC-MS/MS using a Waters Quattro Micro API triple quadrupole mass spectrometer (Milford, MA) interfaced with Acquity UPLC system. Nitrogen was used as nebulizer and argon was used as collision gas. The source temperature was maintained at 120°C, and the desolvation temperature was kept at 450°C. The drying gas (N_2_) was maintained at ca. 800 L/h whereas the cone flow gas was off. The multiplier was set at an absolute value of 650. Online chromatographic separation was achieved using a Supelcosil-LC-18-DB column (33×3.0 mm i.d. 3.0 µm). Gradient elution with methanol and water charged with ammonium acetate and formic acid were employed.

Optimized parameters for GC and GS were determined with individual standard compounds (Avanti Polar lipids, Inc.). For quantification of GCs, the ESI-MS/MS was operated in the multiple reaction monitoring (MRM) mode for monitoring transition pair of the individual protonated precursor ions and their common product ion m/z 264. GS was monitored by mass transition m/z 462.3>282.4. Calibration curves were built for C16, C18 and C24:1 GC using C12 GC as the internal standard. Quantification of GCs with various fatty acid chain lengths was realized by using the curve of each GC species with closest number of chain length. The quantification of GS was based on the curve using C8 GS as internal standard. The linear response for GCs and GS was in the range of 50 pg–25 ng. The extracted spleen and lung samples were suspended in methanol containing internal standard and injected into the LC/MS. The GC and GS levels in the spleen and lung were normalized to tissue weight.

### Analyses of GCase activity

Tissues were collected from saline perfused mice, homogenized in 0.25% Na taurocholate and 0.25% Triton X-100 and their GCase activities were determined fluorometrically [24], [38]. Assay mixtures were incubated in the presence of CBE for 60 min (37°C) to correct for the presence of non-lysosomal GCase and β-glucosidases that cleave 4MU-Glc. WT mice tissues were run in parallel as control. Data were analyzed by Student's t-test.

### Immunoblots

Mouse liver GCase protein detection by immunoblot was as described [36]. The amounts of GCase protein were quantitated with ImageQuant software and normalized to β-actin in the same samples. Midbrain tissues were homogenized in M-PER Mammalian Protein Extraction Reagent and subjected to electrophoresis on NuPAGE 4-12% Bis-Tris gel. The protein was transferred to Hybond™-ECL™ nitrocellulose membrane. GFAP protein in the samples was reacted with anti-GFAP antibody [39]. Anti-active p38 polyclonal antibody was used to detect Phosphorylated p38 (Phospho-p38) in the samples resolved on 10% Bis-Tris gel [19]. The signals were detected with ECL detection reagent (GE Healthcare, Piscataway, NJ) according to manufacturer's instructions.

### qRT-PCR

RNAs in midbrain or brain stem tissues were extracted using TOTALLY RNA kit (Ambion Inc.) and purified with RNeasy Mini kit (Qiagen). Reverse transcription of total RNA (1 µg) for each sample was carried out using RT^2^ First Strand Kit (SA Biosciences). Quantitative RT-PCR (qRT-PCR) was performed on ABI Prism 7000 sequence detection system. The primers for mouse TNFα, IL6, and GAPDH were from SA Biosciences. In PCR mixtures, cDNA aliquots were analyzed using 400 nM of each primer and RT^2^ Real-Time SYBR Green/ROX PCR master mix (SA Biosciences). PCR amplifications were run in duplicate as follows: 2 min at 50°C and 10 min at 95°C, followed by 40 cycles of 15 s at 95°C and 1 min at 60°C. Relative quantification of TNFα or IL6 referenced to internal control, GAPDH, was analyzed by ΔΔCt Analysis. The fold changes of each gene were normalized by WT samples. Three mice from each group were used and the experiments were repeated twice.
